# miRNAs regulate the HIF switch during hypoxia: a novel therapeutic target

**DOI:** 10.1007/s10456-018-9600-2

**Published:** 2018-01-27

**Authors:** Marcin Serocki, Sylwia Bartoszewska, Anna Janaszak-Jasiecka, Renata J. Ochocka, James F. Collawn, Rafał Bartoszewski

**Affiliations:** 1 Department of Biology and Pharmaceutical Botany, Medical University of Gdansk, Hallera 107, 80-416 Gdańsk, Poland; 2 Department of Inorganic Chemistry, Medical University of Gdansk, Gdańsk, Poland; 3 Department of Cell, Developmental and Integrative Biology, University of Alabama at Birmingham, Birmingham, AL USA

**Keywords:** microRNAs, HIFs, Hypoxia, Target protectors, Morpholinos

## Abstract

The decline of oxygen tension in the tissues below the physiological demand leads to the hypoxic adaptive response. This physiological consequence enables cells to recover from this cellular insult. Understanding the cellular pathways that mediate recovery from hypoxia is therefore critical for developing novel therapeutic approaches for cardiovascular diseases and cancer. The master regulators of oxygen homeostasis that control angiogenesis during hypoxia are hypoxia-inducible factors (HIFs). HIF-1 and HIF-2 function as transcriptional regulators and have both unique and overlapping target genes, whereas the role of HIF-3 is less clear. HIF-1 governs the acute adaptation to hypoxia, whereas HIF-2 and HIF-3 expressions begin during chronic hypoxia in human endothelium. When HIF-1 levels decline, HIF-2 and HIF-3 increase. This switch from HIF-1 to HIF-2 and HIF-3 signaling is required in order to adapt the endothelium to prolonged hypoxia. During prolonged hypoxia, the HIF-1 levels and activity are reduced, despite the lack of oxygen-dependent protein degradation. Although numerous protein factors have been proposed to modulate the HIF pathways, their application for HIF-targeted therapy is rather limited. Recently, the miRNAs that endogenously regulate gene expression via the RNA interference (RNAi) pathway have been shown to play critical roles in the hypoxia response pathways. Furthermore, these classes of RNAs provide therapeutic possibilities to selectively target HIFs and thus modulate the HIF switch. Here, we review the significance of the microRNAs on the relationship between the HIFs under both physiological and pathophysiological conditions.

**Electronic supplementary material:**

The online version of this article (10.1007/s10456-018-9600-2) contains supplementary material, which is available to authorized users.

## The different roles of HIF-1, HIF-2 and HIF-3 during hypoxia

Many of the deadliest human diseases including coronary artery disease, stroke, chronic obstructive pulmonary disease and cancer are associated with various levels of hypoxia. Cell survival during hypoxia requires the activation of a number of molecular signaling pathways that stimulate angiogenesis, erythropoietin production and metabolism reprogramming to favor glycolysis, and this biological response is termed the hypoxic adaptive response [1]. Unsuccessful oxygen homeostasis, however, that results from chronic hypoxia, may lead to apoptosis, except in the case of various types of cancer [2]. The hypoxic adaptive response, therefore, is crucial for recovery from stroke or myocardial infarction, whereas re-activation of the hypoxia-related cell death pathway can lead to inhibition of tumor growth. Having the ability to manipulate this pathway, therefore, offers a number of important therapeutic opportunities.

**Fig. 1:**
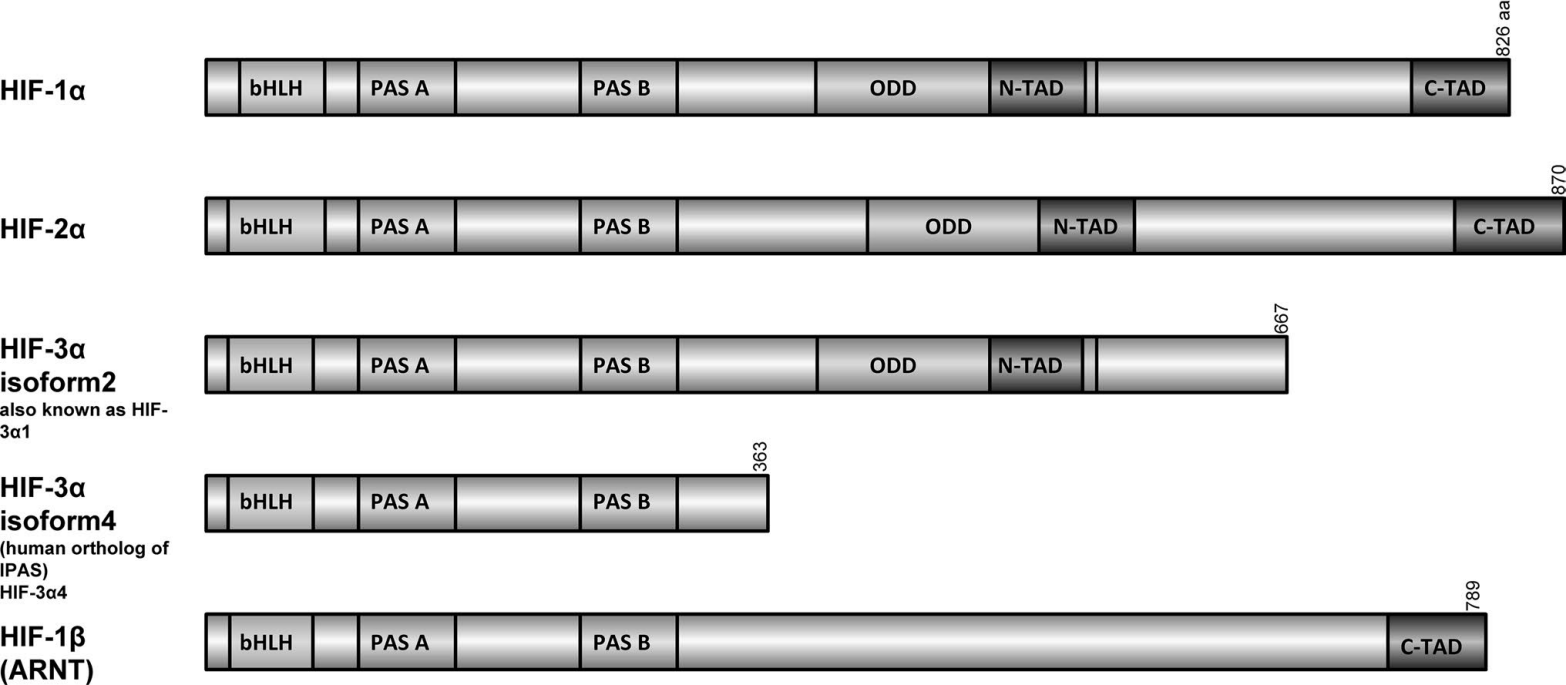
Schematic representation of the HIF subunit domain structures. *ODD* oxygen-dependent degradation domain, *bHLH* basic helix–loop–helix, *PAS* PER–ARNT–SIM, *TAD* transactivation domain, *aa* number of amino acid residues

The activation of the hypoxic adaptive response depends on the function of transcription factors called hypoxia-inducible factors (HIFs). HIFs in response to the unmet oxygen demand of cells activate a number of signaling pathways that are required for cell survival [3]. There are three different HIFs, HIF-1, HIF-2 and HIF-3, that are present in human tissues and are tightly regulated through changes in oxygen tension [4]. HIFs are heterodimers of stable, constitutively expressed β subunits (also called aryl hydrocarbon receptor nuclear translocator or HIF-1β (*ARNT*), HIF-2β (*ARNT2*) and HIF-3β (*ARNTL*) that associate with the hypoxia-induced α subunits, HIF-1α, HIF-2α and HIF-3α [5] (Fig. 1). The amino-terminal end of both α and β subunits consists of basic helix–loop–helix (bHLH) and PAS (PER–ARNT–SIM) domains that mediate heterodimerization and DNA binding [6]. The carboxy-terminal domain of HIF-1α and HIF-2α consists of domains that regulate its stability (the oxygen-dependent degradation domain, ODD) and transcriptional activity (two transactivation domains (TADs), N-TAD and C-TAD [6]. Furthermore, both the C- and N-termini of α subunits have nuclear localization signals (N-NLS and C-NLS, respectively) that direct them to the nucleus [7]. HIF-1α and HIF-2α have 48% amino acid sequence identity and similar protein structures, and they differ mainly within the N-TAD domain [8]. HIF-3α is similar to HIF-1α and HIF-2α in the bHLH and PAS domains, but lacks the C-terminal transactivation domain [2]. Alternative splicing of *HIF3A*, however, as well as utilization of different promoters results in at least four different *HIF3A* mRNA variants that code for six or more isoforms [9]. To date, the most studied HIF-3 variant is the inhibitory PAS domain protein (IPAS), which is a truncated protein [10] that inhibits HIF-1 and HIF-2 activity in cell culture [10]. The other human HIF-3 isoforms, in contrast, were reported to induce gene expression, indicating that that HIF-3 can also be an important transcriptional regulator of hypoxic signaling [11, 12].

## Regulation of HIF stability

All three HIF-α proteins show oxygen-regulated protein stability [5]. During normoxia, posttranslational hydroxylations of alpha subunits occur by hydroxylase enzymes, proline-hydroxylases (PHDs) and factor inhibiting hypoxia-inducible factor-1 (FIH-1; also known as HIF-1α subunit inhibitor, *HIF1AN*). During normoxia, these hydroxylase enzymes target the alpha subunits for polyubiquitination and degradation [13]. The PHD-dependent hydroxylation of specific proline residues in the ODD domains leads to recruitment of the product of the tumor suppressor gene von Hippel-Lindau E3 ligase (pVHL) and other protein cofactors and results in 26S proteasomal degradation of the alpha subunits. The crucial role of pVHL in regulation of HIF homeostasis is illustrated by the fact that mice embryos with inactivation of VHL die in utero due to defects in placental development, whereas heterozygous VHL mice appear phenotypically normal [14].

In order to hydroxylate HIF-α, PHDs require molecular oxygen, 2-oxoglutarate, iron ions (Fe^2+^) and ascorbic acid [15]. Furthermore, PHDs isoforms show different HIF specificity and PHD-2 activity is mainly HIF-1α-specific [16], whereas HIF-2α levels are controlled mainly by PHD-3 [17]. Although the complete ablation of Phd1 and Phd3 in mice had very limited effects on animal development and phenotype, the disruption of the Phd2 gene resulted in a global increase in HIF-α levels and severe placental and heart defects that resulted in embryonic death [18].

The second hydroxylase, FIH-1, regulates the transcriptional activity of the heterodimeric complex by hydroxylation of a single asparagine residue in transactivation domains of the α subunits of HIF-1 and HIF-2 [19]. This posttranslational modification prevents a late key step in the HIF activation process, the dependent recruitment of the co-activators CREB-binding protein (CBP, also known as CREBBP) and p300, which have histone acetyltransferase activity [20]. The FIH-1 activity also requires molecular oxygen. Importantly, FIH-1 preferentially hydroxylates HIF-1α and requires lower oxygen tension to maintain activity than PHD-2 [21]. Interestingly however, the complete impairment of *FIH* gene expression in mice had little to no effect on related HIF signaling, including angiogenesis, erythropoiesis, or development [22]. Hence, further examination of whether FIH-1 regulates HIFα activity in vivo is necessary.

During normoxia, both PHD-2 and FIH-1 are active and maintain low HIF protein levels and transcriptional activity [23]. However, low oxygen tension impairs PHD-2 and FIH-1 activities and leads to the HIF-alpha subunit stabilization. Subsequently, the alpha subunits translocate to the nucleus and after dimerization with the β subunits form transcriptionally active HIF complexes [24]. HIF-1 and HIF-2 mediate the endothelial hypoxic response by binding to the hypoxia response element (HRE) sequences in the promoters of their unique and common target genes and enhance their expression [2]. The HREs consist of an asymmetric E-box motif RCGTG (where R is A or G) [25]. Although HIF-mediated negative transcriptional regulation has been reported [26], it is almost entirely indirect [27]. Finally, the hypoxic recruitment of the CREBBP and p300 to HIF complexes allows an additional level of modulation of HIF transcriptional activity in response to various environmental stimuli [20], whereas the hypoxia-induced sirtuin 1 (SIRT1) selectively deacetylates HIF-1α during hypoxia and leads to increased HIF-1 activity [28].

## HIF target genes

HIF-1 complexes are present in all tissues, whereas HIF-2 is limited to specific cell types that include endothelial cells (ECs), glial cells, type II pneumocytes, cardiomyocytes, kidney fibroblasts, interstitial cells and hepatocytes [29]. In vitro studies show that the efficiency of hydroxylation of HIF-2α, by both the PHDs and FIH-1, is much lower than for HIF-1α, which results in the accumulation and activation of HIF-2α at higher oxygen tensions than HIF-1α [30]. However, although the O_2_ tensions stabilizing the HIF-2α in vitro were often close to the physiological ones observed in tissues (normoxia in situ), HIF-2α was not detected under normoxic conditions in vivo in the organs examined [31, 32].

The genes activated by HIF-1 enhance oxygen delivery to the tissues and/or promote cellular metabolic adaption to the reduced oxygen levels [3]. Indeed, HIF-1, but not HIF-2, is responsible for the transcriptional regulation of the glycolytic pathway enzymes such as phosphofructokinase (*PFK*) and lactate dehydrogenase A (*LDHA*) [33]. Furthermore, HIF-1 induces genes that are involved in pH regulation (monocarboxylate transporter 4 (*MCT4*) and carbonic anhydrase 9 (*CA*-*IX*)); in apoptosis induction (*BCL2*/adenovirus E1B 19 kDa-interacting protein 3 (*BNIP3*) and BCL2/adenovirus E1B 19 kDa-interacting protein 3-like (*BNIP3L/NIX*)) [17]; and in maintaining endothelial homeostasis (endothelial nitric oxide synthase (*NOS3*)) [34]. Another target of HIF-1 during hypoxia is heme oxygenase-1 (*HMOX1*) that has pro-angiogenic activity [35], whereas HIF-2 stimulates the expression of matrix metalloproteinases (MMP) 2 and 13 and the stem cell factor OCT-3/4 [17]. Furthermore, silencing HIF-2α in human microvascular endothelial cells (hMVECs) exposed to prolonged hypoxia indicated that the transcriptional activity of this HIF isoform specifically inhibited endothelial sprouting [36, 37]. This specific activation of HIF target gene expression was proposed to be independent of selective HRE-binding and relied on the non-equivalent C-terminal regions of these factors’ alpha subunits [38].

Common targets of HIF-1 and HIF-2 include the vascular endothelial growth factor A (*VEGFA*), the glucose transporter 1 (*GLUT1*) and erythropoietin (*EPO*) [2]. However, the latter one is considered to be mainly a HIF-2 target gene [29]. Erythropoiesis relies on iron availability, and HIFs regulate the expression of several genes involved in iron homeostasis, including transferrin [39]. Importantly, a conserved iron response element (IRE) identified in the 5′UTR of HIF-2α mRNA was shown to mediate HIF-2α protein translation during increased iron availability, thereby providing a functional link between hypoxia-related erythropoiesis and iron homeostasis [40, 41].

It needs to be stressed that for many genes, the HIF selectivity is highly cell-type-specific [42]. The differences in tissue-specific effects of HIF-1 and HIF-2 on target genes have been proposed to be conferred by cooperation of the HIF-alpha TAD domains (particularly the N-TAD) with other transcription cofactors, possibly in a tissue-specific manner [43]. To date, members of Ets family transcription factors have been shown to support HIF-2α-related hypoxic gene induction [43–45]. Importantly, although endogenous HIF-2 alone was shown to be inefficient in compensating for HIF-1 function during hypoxia [46], it has been also reported that in the absence of HIF-1, the exogenous HIF-2 was able to activate HIF-1-specific genes and vice versa [46, 47]. Furthermore, in HIF-2α knockdown mice, HIF-1α regulates the expression of genes that are normally regulated by HIF-2α [48].

## HIF knockout/knockdown animal models

The essential role of the HIF pathway in the cellular and developmental aspects of oxygen homeostasis has been also confirmed in animal knockout/knockdown models. Furthermore, the results of HIF-specific knockouts and knockdowns stress the different biological and transcriptional functions of these factors as well as their complex physiological functions [49]. The complete impairment of HIF-1α expression in mouse tissues results in developmental arrest and early embryonic lethality that manifested as defects in capillarization and a complete lack of cephalic vascularization and a greatly increased degree of hypoxia [50, 51]. Similarly, mice with complete deficiency of HIF-2α were embryonic or perinatal lethal, with embryos that develop severe vascular defects [52, 53]. However, by crossing mice in different genetic backgrounds, a small fraction of viable Hif-2α-null adult mice were obtained [54]. These mice exhibit multi-organ dysfunction associated with increased oxidative stress levels, suggesting that HIF-2α is a crucial factor for regulating the expression of antioxidant enzymes [54]. Importantly, HIF-2α knockout mice did not show impaired blood vessels formation by vasculogenesis, but later fusion and assembly, suggesting that HIF-2α activity is critical for the remodeling of the primary vascular network into a mature hierarchy pattern [52]. Hence, both HIF-1 and HIF-2 play essential roles, but cannot fully compensate for each other during embryonic development [2]. Taken together, the data imply the necessity of the transition from HIF-1- to HIF-2 signaling during embryonic vascular development in order to adapt it to the increase in oxygen tension as the vasculature develops. However, the mechanism for the developmental switch from HIF-1 to HIF-2 remains unknown.

In contrast to the lethal HIF-1α and HIF-2α mice models, the HIF-3α-depleted mice were viable, but displayed impaired heart and lung development during the embryonic and neonatal stages [55]. The loss of HIF-3α in mouse pulmonary endothelial cells results in impaired angiogenesis both in normoxic and in hypoxic conditions, supporting the direct transcriptional involvement of HIF-3 in hypoxic signaling [56].

Since the analysis of a biological function of HIF-1α and HIF-2α after birth is hampered by the null mice lethality, heterozygous mice and cell-type-specific HIF-1α and HIF-2α knockouts/knockdowns remain the main tools for studying the consequences of these HIF functional impairments [57]. Mice that are HIF-1α heterozygous develop normally and are indistinguishable from their wild-type littermates in normoxia [50, 58]. Upon exposure to chronic hypoxia, however, these mice develop ventilatory abnormalities [59]. The wild-type mice, however, were more prone to develop polycythemia, right ventricular hypertrophy, pulmonary hypertension and pulmonary vascular remodeling during chronic hypoxia [58]. Furthermore, numerous studies that examined the consequences of loss of HIF-1α in different tissues demonstrated that this transcription factor’s function is not limited to hypoxic adaptation, but also modulates other critical physiological functions [57] including immune responses [60], chondrogenesis [61] and osteoblast development [62]. Importantly, mouse endothelial cells (ECs) lacking HIF-1α display reduced proliferation and decreased tubular network formation during hypoxia, and loss of HIF-1α in these cells leads to impairment of a hypoxia-driven VEGF autocrine loop that is crucial for tumorigenesis [63].

HIF-2α heterozygous knockdown mice show normal development similar to HIF-1α heterozygous knockdown [64]. Importantly, upon exposure to hypoxia, the HIF-2α knockdown mice displayed impaired neovascularization in the retina [64]. Furthermore, the reduced HIF-2α expression in these animals results in higher susceptibility to renal injury, and elevation of endothelia-related oxidative stress response signaling [65]. In contrast, heterozygosity for HIF-2α appeared to be protective against pulmonary hypertension and right ventricular hypertrophy induced by chronic hypoxia [49], similar to HIF-1α heterozygosity. Although HIF-2α knockdown mice are anemic, the anemia did not affect their susceptibility to ischemia [65]. Importantly, the acute deletion of HIF-2α resulted in anemia associated with decreased levels of circulating Epo and thus confirmed that this gene is a physiological target of HIF-2α in adult mice [66]. Interestingly, the specific deletion of HIF-2α in murine endothelial cells did not affect mouse development, but resulted in increased vessel permeability, aberrant endothelial cell ultrastructure, cell adhesion and pulmonary hypertension [67]. HIF-2α-depleted ECs displayed defective hypoxic induction of target genes that likely contribute to these phenotypes [67]. Importantly, the VEGF expression was unaffected in HIF-2α-depleted ECs, suggesting that this gene is a HIF-1-specific target in these cells [4, 67].

## HIF effects on angiogenesis

Taken together, the results of HIF-specific knockouts imply different physiological functions of HIF-alpha isoforms with HIF-1 promoting initial angiogenesis, and HIF-2 and HIF-3 instructing further development of the vascular network in a tissue-specific manner. Overall, the studies highlight the necessity of maintaining a proper HIF balance during development and during pathological conditions.

**Fig. 2:**
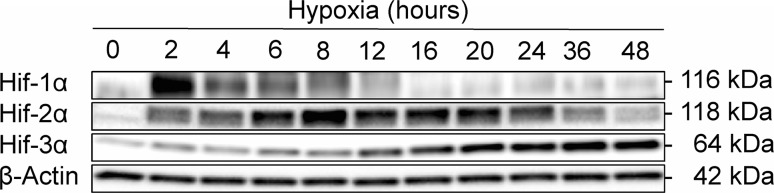
Hypoxia (0.8% O_2_) induces dynamic changes in the protein expression profile of the Hif-1α, Hif-2α and HIF-3α in primary HUVECs (pooled from 10 donors, passage 3). The protein levels of were detected with SDS-PAGE and Western blot and normalized to β Actin levels. Two individual samples (20 µg of total protein per lane) were tested for each time point, and the experiments were repeated twice. The primary antibodies used were: Hif-1α (Abcam ab16066, diluted at 1:2000); Hif-2α (Abcam ab199, diluted at 1:1000), Hif-3α [12] (Sigma AV39936, diluted at 1:1000), beta Actin (Abcam ab1801, diluted at 1:1000)

Consequently, in endothelium exposed to hypoxic stress, HIF-1 governs the initial/acute adaptation to hypoxia and promotes the formation of a primitive vascular network, whereas HIF-2 and HIF-3 expressions begin after more prolonged oxygen depletion and allow maturation and stabilization of this vasculature [2]. Furthermore, the formation of HIF-1-dependent vasculature leads to increased perfusion and thus increases in oxygen concentrations that may lead to HIF-2 accumulation, which is less prone to oxygen-dependent destabilization. Hence, in endothelial tissues, there is a switch in HIF signaling during prolonged hypoxia. The HIF-1 levels are reduced during this period, whereas HIF-2 and HIF-3 are induced and accumulate. As shown in the primary human endothelial cells (HUVECs) in Fig. 2, HIF-1 governs the HRE-dependent cellular responses during first 8 h of hypoxia, then HIF-2 becomes main regulator for the next 24 h. Importantly, both HIF-1 and HIF-2 are highly expressed at the 8-h time point, showing that in cell culture, the HIF switch occurs gradually within hours. Furthermore, the inability to reduce the HIF-1 levels during prolonged hypoxia leads to cell death, making this a potential therapeutic target in cancer. Therefore, the described HIF switch is crucial for endothelial adaptation to continuous oxygen depletion. Passing the signal from HIF-1 to HIF-2 and eventually to HIF-3 allows the cells the ability to promote angiogenesis and long-term survival.

To date, only a few studies have evaluated the genome-wide effects of HIFs in vitro in human pulmonary artery ECs and HUVECs during 24 and 48 h of hypoxic exposure [68, 69]. Furthermore, there are no reports that focus on the transcriptional consequences of acute hypoxia (less than 12 h) and only one that followed the effects of prolonged hypoxia (14 days) in human microvascular endothelial cells (hMVECs) [36]. However, the genome-wide analysis of human colon carcinoma cells compared the transcriptome changes observed during acute hypoxia during chronic hypoxia and found dramatic differences in the hypoxia transcriptional response [70], with similar results also described in [71]. Acute hypoxia affected the expression of only 196 genes, whereas chronic hypoxia resulted in changes in 4149 transcripts [70]. Interestingly, 144 genes were common for these two types of hypoxia [70]. These large-scale changes in transcriptome are presumably required to adapt the cells to chronic hypoxia. Although this differential expression mainly results from the activation of downstream transcriptional effects during hypoxia, it is also a result of the different HIF isoforms.

To take advantage of regulating angiogenesis as a possible therapeutic intervention, it is important to understand the molecular pathways governing endothelial adaptation to hypoxia and especially the HIF switch from HIF-1 to HIF-2. It should also be emphasized that the distinction between acute and prolonged hypoxia is rather arbitrary and does not necessarily reflect the pathological conditions. For example, in vivo tissue oxygen tension can remain low for prolonged periods and often switches (in tumor tissues) or cycles between normoxic and hypoxic levels. Finally, most of the studies relate hypoxic conditions to normoxia defined as atmospheric oxygen pressure (above 20%), and this is many fold higher than physiological normoxia in situ.

Although HIF regulation during normoxic conditions is carefully controlled by the hydroxylases described above, how HIF activity and angiogenesis are controlled during hypoxia is less clear. The mechanisms underlying the HIF switch and related HIF-1 level reductions remain poorly understood. Importantly during prolonged hypoxia, the HIF-1 levels are reduced, despite the lack of oxygen-dependent degradation.

## Other mechanisms that regulate the HIF switch

SART1, the human homolog of murine hypoxia-associated factor (HAF), is a transcription factor that has been proposed to be a crucial mediator of the HIF switch. The SART1/HAF protein selectively binds and degrades HIF-1α while promoting HIF-2α stability and transactivation [72]. The SART1 levels first decrease during acute hypoxia and gradually increase with prolonged hypoxia exposure that destabilizes HIF-1 and stabilizes HIF-2 [73]. Therefore, SART1 provides an elegant mechanism for the protein stability-based switch from HIF-1 to HIF-2 signaling [2]. Another mechanism that contributes to the HIF switch is based on the Hsp70/CHIP (carboxyl terminus of Hsp70-interacting protein) complex that during prolonged hypoxia selectively ubiquitylates HIF-1α but not HIF-2α and leads to proteasomal degradation [74]. A third mechanism potentially involved in the protein stability-based switch from HIF-1 to HIF-2 signaling relies on the interplay between the receptor for activated kinase C1 (RACK1) that promotes degradation of HIF-1α and the heat shock protein 90 (Hsp90) that stabilizes the α subunit [75, 76]. RACK1 promotes PHD-/VHL-independent proteasomal degradation of HIF-1α and competes with Hsp90 for binding to the PAS-A domain of HIF-1α [75, 76]. RACK1 binds to Elongin-C and promotes ubiquitination of HIF-1α [75, 76]. Also, human double min 2 (hdm2) has been reported to induce HIF-1α proteasomal degradation in a p53-dependent manner [77].

Another example is the Kruppel-like Factor 2 (KLF2), which is strongly expressed in endothelial cells and necessary for normal vessel formation [78]. KLF2 has been shown to selectively promote Hif-1α degradation during hypoxia in a von Hippel-Lindau-independent, but proteasome-dependent manner through disruption of the interaction between Hif-1α and Hsp90. Interestingly, Klf2 has no effect on Hif-2α protein stability [79]. Furthermore, as suggested by studies in the conditional PHD2-deficient mice [80, 81], the HIF-1-dependent induction PHD3 that is able to govern HIF-2α degradation both in normoxia and in hypoxia [15] could be a limiting factor for HIF-2α levels during hypoxia. Hence, HIF-1 through induction of PHD3 could prevent premature HIF-2 accumulation during acute hypoxia and thus provide an addition mechanism for the HIF switch.

Clearly, further studies will be necessary to clarify whether these above mechanisms provide the selection between HIF-1 and HIF-2. The above-discussed mechanisms contributing the HIF switch strongly rely on stabilizing/destabilizing α subunits and have been described mainly in cancer cells and animal models. Determining whether similar regulatory mechanisms also take place in normal human endothelia from different vascular beds remains an open question.

The stability of the mRNA of HIF-α subunits also differs during hypoxia following the HIF switch. Indeed, the *HIF1A* mRNA after the initial induction is reduced to normoxic levels, whereas *EPAS1* and *HIF3A* mRNAs stabilize and accumulate during prolonged hypoxia [12]. Hence, the selective reduction of the protein output from existing HIF-α subunits transcripts provides a novel mechanism for controlling HIF expression and their related signaling during hypoxia.

## Role of miRs in regulating HIF signaling

microRNAs (miRNAs, miRs) are nonprotein coding RNA molecules that govern mRNA stability and translation by binding to the 3′UTR of the mRNA, resulting in reduced protein levels. microRNAs are encoded in both introns and intergenic clusters as short inverted repeats and have a double-stranded RNA (dsRNA) stem loop that is about 70 bp long [82]. These primary microRNAs (pri-miRNA) genes are transcribed by RNA polymerase II into long primary miRNA (pri-miRNA) transcripts [83]. The double-strand-specific ribonuclease Drosha-DGCR8 complex processes the pri-miRNA transcripts to the precursor miRNA (pre-miRNA) stem loop structures [83]. The pre-miRNAs are next transported to the cytoplasm where they are cleaved by the Dicer RNAase III endonuclease to produce the mature 21–23 nucleotide miRNAs [83, 84]. Mature miRNAs are incorporated into the Argonaute-containing silencing complexes called the miRNA ribonucleoprotein complex (miRNP) and down regulate specific target mRNAs via either decreasing the transcript levels or by translational repression [85, 86]. They act as adaptors for the miRNA-induced silencing complex (RISC) to initiate mRNA decay and thus reduce protein output. Mature miRNAs recognize their target mRNAs through base-pairing interactions between nucleotides numbers 2 and 8 of the miRNA (the seed region) and the complementary nucleotides in the 3′-untranslated region (3′-UTR) of the mRNAs [87].

Given that miRNAs reduce the protein output from existing transcripts, they are the perfect candidates for controlling HIF expression during hypoxia. Hence, during early hypoxia, specific temporal changes of miRNA levels may contribute to HIF-1 accumulation and the maintenance of the steady-state levels of HIF-2 and HIF-3. Whereas during prolonged hypoxia, the miRNA expression is changed to help to maintain low HIF-1 function and maintain elevated HIF-2 and HIF-3 levels. Hence, miRNAs have the ability govern the hypoxic HIF switch in human endothelia.

Although hypoxia and ischemia change the expression profiles of many miRNAs [88], a functional role for a limited number of these so-called hypoxamiRs [89] has been demonstrated and number of these that affect HIF expression have been identified [90]. Furthermore, despite the growing number of known HIF-related miRs, a large number of studies have focused on cancer cell lines only and often ignore endothelial cells and the role of hypoxia-induced miRNAs. Importantly, different human tissues display a different spectrum of hypoxic responses, including changes in HIF expression that might result from tissue-specific miRNA expression. Indeed, many miRNAs are expressed in tissue- and age-specific patterns [91]. The results are also often conflicting regarding the changes in miRNA levels during a time course of hypoxia as well. Interestingly, reports in cancer cells have shown that hypoxia results in reduced expression of genes involved in miRNA biogenesis, including *DICER* [92] and *DROSHA* [93]. Simultaneously, however, they have increased expression of genes responsible for miRNA function like *EIF2C4* that encodes a crucial component of the RISC complex Argonaute 4 (AGO4) [92]. In human primary endothelial cells (HUVECs), hypoxia reduces mRNA and protein expression of many miRNA-processing subunits including DICER [94]. Hypoxic reduction of DICER results from activity of the oxygen-dependent H3K27me3 demethylases lysine demethylase 6A and 6B (KDM6A/B), which suppress the *DICER* promoter and lead to reduced miRNA biogenesis during hypoxia [95]. Although one could expect a general dramatic reduction in mature miRNA levels during hypoxia along with an increase in mRNAs stability, analysis of pri-miRNAs and mature miRNAs levels during hypoxia has shown this not to be the case and suggests that additional mechanisms are involved in maintaining hypoxic miRNA expression [92]. Alternatively, it could suggest that although expression of proteins responsible for miRNA biogenesis is reduced during hypoxia, their levels are still maintained. Furthermore, Dicer activity is not required for maturation of all miRNAs [96].

The majority of the studies conducted so far have considered simplified models, where a single miRNA effects were analyzed in the context of one or more mRNA targets. Although these studies advance our understanding of miRNAs function as cellular regulators, they overlook the important fact that a single mRNA can be regulated by the simultaneous coordinated actions of a number of different miRNAs. For example, the *HIF1A* 3′UTR is 1174 base long, while the miRNA seed sequence is usually 6–8 bases. Hence, *HIF1A* mRNA can bind a combination of miRNAs simultaneously, and these pools of miRNA will further determine this mRNA’s translation and stability.

At present, and summarized in Supplemental Table 1, 40 miRNAs have been identified that modulate HIF expression. Conversely, HIF-1 promotes the expression of several hypoxamiRs including miR-210 [97], miR-146a [98], miR-145 [99], miR-382 [100], miR-191 [101], miR-363 [102], miR-421 [103] in tumor cells, miR-204 in neuronal cells [104], miR-30a and miR-21 in cardiomyocytes [105, 106], miR-687 in embryonic kidney cells [107], miR-155 in intestinal epithelial cells [108], and miR-429 [109] and miR-19a [110] in endothelial cells. It has to be stressed that among miRNA that were reported to directly target HIF mRNAs in cancer cells, few could be classified as hypoxamiRs and validated in normal endothelial cells (combined in Table 1). Below, we discuss hypoxamiRs and miRs and how they may contribute to the HIF switch.

**Table 1:** MicroRNAs involved in regulating HIFs and HIF regulatory gene levels in ECs

miRNA	Cell type	Impact of hypoxia on miRNA expression	miRNA target (s) (direct or indirect*)	Investigated processes	References
**miR-18a**	Choroidal endothelial cells	Upregulated	*HIF1A*	Proliferation migration	[116]
**miR-107**	Endothelial progenitor cells—EPCs	Upregulated	*ARNT*	Differentiation	[121]
miR-135b	HUVECs	Upregulated	*HIF1AN*	Angiogenesis	[124]
			HIF1A*		
**miR-155**	Mouse skin endothelial SENDs cells and HUVECs	Upregulated	*HIF1A*	Angiogenesis hypoxia	[108]
**miR-199a**	Endometrial stromal cells; endothelial EA.hy926 cells	Not shown	*HIF1A*	Angiogenesis	[123]
miR-200b	HMVECs	Downregulated upregulated	*ETS1*	Angiogenesis hypoxia	[135, 136]
	HUVECs		*KLF2*		
**miR-210**	HUVECs	Upregulated	*EFNA3*	Angiogenesis hypoxia	[131, 132, 150]
			*HIF3A*		
miR-424	HUVECs, hMVECs, hBOECs and hMBECs	Upregulated	*CUL2*	Angiogenesis	[137]
			HIF1A*		
**miR-429**	HUVECs	Upregulated	*HIF1A*	Hypoxia	[12, 109]
			*HIF3A*		
**miR-433**	HUVECs	Downregulated	*HIF1A*	Proliferation and migration	[138]

miRNAs proven to directly bind HIF mRNAs are in bold, and indirect effects are marked with “*”

*ARNT* aryl hydrocarbon receptor nuclear translocator, *CUL2* cullin-2, *EFNA3* ephrin A3, *EGLN1* prolyl hydroxylase domain-containing protein 2 (PHD2), *ETS1* ETS Proto-Oncogene 1, Transcription Factor, *HIF1A* hypoxia-inducible factor 1-alpha, *HIF1AN* hypoxia-inducible factor 1-alpha inhibitor, *HIF3A* hypoxia-inducible factor 3 alpha, *KLF2* Kruppel-like factor 2

## Role of miRs in regulating the HIF switch

The miR-17 family includes miR-17, miR-18a/b miR-20a/b, miR-93 and miR-106a/b. With the exception of miR-93, these miRNAs are produced from several miRNA gene clusters, which apparently arose from a series of ancient evolutionary genetic duplication events, and also include members of the miR-19 and miR-25 families [111]. In humans, the miRNA constituents of the clusters are overexpressed in multiple cancer types. Importantly, some of these family members have been proposed to be hypoxamiRs [112–115]. miR-18a expression is markedly upregulated after 24 h hypoxia in human choroidal endothelial cells and can directly target *HIF1A* mRNA [116]. Hence, the hypoxic induction of miR-18a may allow HIF-1α level decreases and thus contribute to the HIF switch. Although the expression changes of other miR-17 family members (miR-17, miR-20a and miR-20b) were reported to be involved in the HIF-related response during hypoxia in cancer cells [114, 117] and in mouse pulmonary artery smooth muscle cells (PASMC) [118], the exact role of these miRNAs needs to be confirmed during hypoxia in human endothelia.

Deng and coworkers identified miR-103/107 as directly targeting HIF-1β subunits in rat PASMCs [119]. They showed that hypoxia reduces miR-103/107 levels in PASMCs and leads to upregulation of HIF-1β, but not HIF-1α [119]. This report confirms a previous study of Yamakuchi and coworkers who showed in human colon cancer cells that miR-107 decreases hypoxia signaling by suppressing the expression of *ARNT1* [120]. Furthermore, in human colon cancer specimens, expression of miR-107 was controlled via p53 and inversely associated with the expression of HIF-1β [120]. In contrast, another study showed that miR-107 was upregulated in hypoxia in rat endothelial progenitor cells and prevents their differentiation via its target HIF-1β [121]. miRNA-mediated beta subunits suppression has been shown to limit HIF-1 activity and angiogenesis and provides a novel, alpha subunit-independent mechanism for HIF signaling regulation. However, the physiological role of this regulation as well as the miRNA specificity against other HIF beta isoforms requires further study.

The polymorphism in the miR-199a target site in HIF1A sequence is associated with pancreatic ductal adenocarcinoma risk [122], whereas forced overexpression of miR-199a in endometrial stromal cells (ESCs) attenuated HIF1A’s angiogenic potential during hypoxia via targeting both VEGFA and HIF1-α in these cells [123]. Unfortunately, however, the changes in physiological levels of miR-199a under normoxia and hypoxia in these cells were not determined [123]. Therefore, the possible physiological effects of miR-199a on HIF-1α in ECs will require further studies.

## Exosomal hypoxamiRs

An exosomal miR-135b formed during hypoxia in multiple myeloma cells directly suppresses *HIF1AN* in HUVECs and leads to increased HIF-1 activity and angiogenesis [124]. This exosomal miR-135 provides an interesting example of potentiation of HIF-1 activity in cell-to-cell transfer. Furthermore, by suppressing *HIF1AN*, miR-135b may allow for a sustained hypoxic response, in spite of the fact that the oxygen levels were partially restored. Exosomal miRNAs that stimulate HIF signaling may be involved in controlling hypoxic response on a larger scale than previously recognized. Furthermore, a recent study found that exosomes derived from human hypoxic oral squamous cell carcinoma (OSCC) cells increased the migration and invasion of OSCC cells in HIF-1α- and HIF-2α-dependent manner and contained high levels of miR-21 [125]. A positive feedback loop between miR-21 and HIF-1 activity has also been recently reported in human cardiomyocytes, where hypoxia induces miR-21 expression [106]. HIF-1 transcriptionally enhances miR-21 promoter activity, and miR-21 stimulates HIF-1α expression and modulates the PTEN/Akt pathway [106]. The miR-21 presence in exosomes suggests that this miR-21 can participate in activation of the hypoxic response between cells, including endothelia, through potentiating HIF-1 signaling. However, further studies will be necessary to validate this possibility.

## Other hypoxamiRs

miR-155 is upregulated by hypoxia in human epithelial colorectal adenocarcinoma cells (Caco2), human endothelia and in the mouse intestine [108, 126]. It contributes directly through binding to *HIF1A* mRNA to a decrease in the levels of HIF-1α mRNA and protein, and to a decrease in transcriptional activity [108]. A role for HIF-1α in the induction of miR-155 during hypoxia has been confirmed [108]. Thus, miR-155 induction commits to an isoform-specific negative feedback loop for HIF-1α activity during prolonged hypoxia [108], establishing miR-155 as an important part of the HIF switch.

miR-210 is the most consistently and significantly induced miRNA during hypoxia. It is also unique in that it is induced in almost all cell lines [127]. The expression of this miRNA is regulated by both HIF-1α [128] and HIF-2α [129]. Overexpression of miR-210 in HUVECs enhances *VEGFA* and vascular endothelial growth factor receptor-2 (*VEGFR2*) expression and thereby promotes angiogenesis [130]. Furthermore, recent studies in human chondrocytes [131] and hepatocellular carcinoma cells [132] confirmed that miR-210 directly targets the mRNA of *HIF3A* and suppresses this HIF protein expression. Hence, miR-210 could contribute to the HIF switch between HIF-1/HIF-2 and HIF-3. During acute hypoxia, miR-210 levels are induced by HIF-1/HIF-2 to prevent HIF-3 accumulation, while during chronic hypoxia HIF-1/HIF-2 levels decline and lead to the reduction of miR-210 and subsequent HIF-3 signaling. However, this hypothesis requires further validation.

Recently, another very interesting example of a positive feedback loop indirectly controlling HIF-1 was proposed for miR-147a in human cervical cancer (HeLa) cells, where miR-147 reduced the levels of a dominant-negative isoform of HIF-3 [133]. Moreover, miR-147a is hypoxia-induced and targets HIF-3, a dominant-negative regulator of HIF-1, and thus miR-147a in turn stabilizes and accumulates HIF-1 [133]. Although these studies provide very novel mechanism contributing to HIF switch, these studies will require confirmation in human endothelial cells.

In mice, the levels of the miR-200 family members miR-200b, miR-200c, and miR-429 increase during ischemic preconditioning and target PHD-2, leading to accumulation of HIF-1α [134]. However, Chan and coworkers demonstrated in hMVECs (human microvascular endothelial cells) that miR-200b is downregulated by prolonged hypoxia (24 h), and thus, the levels of its targets, the pro-angiogenic genes, are induced [135]. In contrast, our studies in HUVECs indicate that the physiological induction of miR-200b during acute hypoxia (4 h) leads to direct Klf2 downregulation and subsequent stabilization of HIF-1 signaling [136]. Furthermore, we demonstrated that miR-429 (clustered along with miR-200a and miR-200b on chromosome 1p36) has the opposite function. Although miR-429 is upregulated during acute hypoxia in primary HUVECs by HIF-1, the HIF-1α levels are negatively regulated by miR-429, establishing a negative regulatory feedback loop [109]. Furthermore, the temporal changes in expression of miR-429 facilitate the HIF switch through the specific reduction of HIF-1 levels and subsequent stabilization of HIF-3 during prolonged hypoxia in human endothelium [12]. This suggests that miR-429 may regulate the transitional switch between HIF-1, HIF-2 and HIF-3 responses in HUVECs under chronic hypoxia by attenuating HIF-1 responses and by delaying the onset of *HIF3A* message stability. However, this hypothesis requires validation in other types of human ECs.

Another elegant miRNA-dependent mechanism of miRNA-related positive HIF-1α stability regulation in human ECs (including HUVECs and hMVECs) during hypoxia is provided by miR-424. microRNA-424 is differentially increased in ECs exposed to hypoxia and targets cullin 2 (CUL2), which is critical to the assembly of the ubiquitin ligase system and leads to the stabilization of HIF1-α [137]. Importantly, the rodent homolog of human miR-424, mu-miR-322, has a similar impact on HIF-1α in experimental models of ischemia [137]. Another miRNA, miR-433, is downregulated in hypoxia-exposed HUVECs and normally directly targets HIF1-α. Hence, the hypoxic reduction of this miRNA could promote HIF-1 signaling [138]. However, since miR-433 was reduced up to 48 h of hypoxia [138], further studies are required to access physiological consequences of this miRNA modulation for the HIF switch.

Although no miRNA has been proposed to directly target HIF-2 levels in human endothelium to date, recent work indicates that miR-588 in neuroblastoma stimulates HIF-2α expression through interaction with 5′ UTR of *EPAS1* mRNA [139]. miR-558 directly binds with its complementary site within this 5′-UTR and facilitates the binding of AGO2 to the eukaryotic translation initiation factor 4E (eIF4E) binding protein 1 and results in increased eIF4E enrichment and HIF-2α translation [139]. Furthermore, miR-17, and miR-18b have been postulated to directly reduce *EPAS1* in human macrophages [140]. Interestingly, a functional binding site of miR-145 in the 3′-untranslated region (3′-UTR) of *EPAS1* mRNA was also confirmed in neuroblastoma cells [141]. Overexpression or knockdown of miR-145 altered both the mRNA and protein levels of HIF-2α and its downstream genes in normoxic conditions, supporting the view that all three HIFs are regulated either directly or indirectly by miRNAs.

## Long noncoding RNAs and HIF regulation

Another group of noncoding RNAs that can be modulated by hypoxia and control HIF signaling is the long noncoding RNAs (lncRNAs). lncRNAs are defined as nonprotein coding transcripts longer than 200 nucleotides [142]. lncRNAs can carry biological functions through chromatin modification, genomic imprinting, and transcriptional interference and activation [142]. To date, a large number of HIF-related lncRNAs have been identified in cancer cells (reviewed in [143]), and here we focus on those that may also contribute to the HIF switch. For example, 5′aHIF1α and 3′aHIF1α lncRNAs are two antisense transcripts that are transcribed from the 3′-untranslated region and 5′-promoter region of the sense *HIF1A* mRNA [144]. These aHIF1α lncRNAs are induced under hypoxia in cancer cells to suppress *HIF1A* mRNA expression [145], thus providing for a HIF switch negative feedback loop. However, although aHIF1α is expressed in many cancers [144], it is unclear how important this is in non-cancerous cells. Other studies in cancer cells have shown that HIF-1α-induced lincRNA-p21 bind HIF-1α and VHL and thus disrupt the VHL–HIF-1α interaction and promote HIF-1α accumulation [146]. Furthermore, the long intergenic noncoding RNA, the regulator of reprogramming (linc-RoR), was increased in hypoxic regions within tumor cell xenografts in vivo and shown to target miR-145 [147]. Thus, linc-RoR could affect either HIF-1 or HIF-2 signaling. Very recently, a study of Lo Dico and coworkers reported that miR-675-5p embedded in hypoxia-induced long noncoding RNA H19 plays a mandatory role in establishing the hypoxic response and in promoting hypoxia-mediated angiogenesis in human glioma cells [148]. In these studies, the direct interaction of miR675-5p, *HIF1A* mRNA and the RNA Binding Protein HuR was also proven [148]. A recent study by Cai and coworkers identified LINC00152 to specifically reduce miR-138 levels and thus stabilize HIF-1α in gastric and hepatocellular cancer cells [149]. The studies discussed above suggest that lncRNAs can affect HIF signaling not only directly but also through miRNA modulation. However, further clarification is needed to establish if these miRNAs-lncRNAs regulations take place in normal human endothelia.

## Unanswered questions

The discussed studies highlight the biological importance of the HIF signaling transition both during development and in pathological conditions. Our understanding, however, regarding the posttranscriptional and posttranslational mechanisms governing this transition in normal human endothelia is rather limited. Although numerous posttranslational interactions have been proposed to explain hypoxic HIF-1α destabilization, very few of these studies were validated in ECs, and their impact on HIF-1α levels and the related consequences mostly remain unknown.

**Fig. 3:**
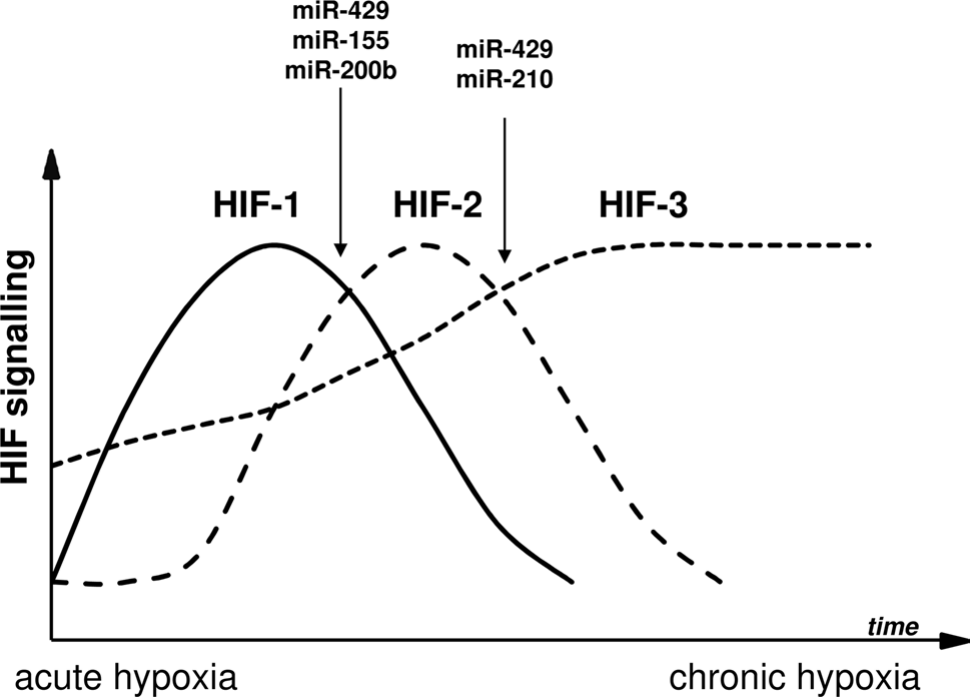
Schematic representation of the miRNA role in the HIF switch regulation

Similarly, while cancer research studies have resulted in the identification of a large number of miRNAs involved either directly or indirectly in controlling HIF expression [151], the functional effects of a small percentage of these miRNAs have been validated in human endothelial cells. Several miRNAs including miR-155 and miR-429 are believed to be involved directly in the HIF switch (Fig. 3). These two miRNAs are induced through HIF-1 and reduce HIF-1α levels, and in doing so, provide a mechanism for establishing the transition to HIF-2 and HIF-3 signaling. The hypoxic changes in both miR-210 and miR-429 also provide a mechanistic explanation for HIF-3α accumulation during chronic hypoxia. The miR-147a-based positive feedback mechanism controlling the downregulation of the dominant-negative isoform of HIF-3 could also be an important part of this regulation. Furthermore, miR-558 provides the very interesting possibility for direct upregulation of HIF-2α levels, but this needs to be verified.

The majority of studies have focused on HIF-1′s role and do not consider the consequences of hypoxia-related miRNA-initiated alterations of HIF-2 and HIF-3 activity. Furthermore to date, only a few miRNAs have been shown to control HIF-2α and Hif-3α expressions. On the other hand, since the HIF switch is a dynamic process, more attention should be paid to follow the temporal miRNA and HIF level alternations during hypoxia. Most of the previous studies have limited their focus to assessment of miRNA/mRNA and protein changes in chronic hypoxia. Although these reports increase our understanding of the miRNA-related aspects of the hypoxic response, the lack of data relating to the temporal miRNA changes with the respective HIF levels limits their utility in evaluating the ncRNAs effects on the HIF transition. Furthermore, since most of current miRNA studies followed their effects on HIF levels in a single type of ECs, it is difficult to distinguish between miRNA’s universal endothelial effects and the tissue-specific ones. Finally, although due to experimental limitations, usually the impact of only one miRNA on one particular HIF level is considered. The most plausible scenario would assume that in vivo the HIF mRNA stability is simultaneously and cooperatively governed by unique sets of miRNAs. How we will be able to untangle this complex mechanism of regulation remains an open question. Finally, although animal models are very critical for determining both the physiological and pathological roles of HIF signaling, the discrepancy in miRNA target selection between human and other species often hampers their translational usefulness. A plethora of different miRNA species are predicted to have different target sites in human and rodents’ 3′UTR of HIF mRNAs, and miRNA site conservancy is a rather poor predictor of their functionality since even a one base change within the target site can impair miRNA function [152]. Furthermore, the most commonly implemented models to study the hypoxic response in human ECs in vitro do not reflect the physiological (normoxia in situ) and pathological conditions in vivo *(*e.g., acute hypoxia, persistent hypoxemia and intermittent hypoxia*)*. Thus, the miRNA organism specificity limitations highlight the need for validation of miRNA–HIF interactions in experimental models to normal human ECs and in human pathophysiology.

To date, many efforts have been made to identify small molecule inhibitors that target the HIF pathway (reviewed in [153]). However, the majority of HIF inhibitors identified so far are based on the evaluation of HIF-1 activity in cultured cancer cell lines. HIF-1 inhibitor activity includes affecting HIF-1α protein levels, HIF-1 dimerization, HIF-1 DNA binding and HIF-1α transcriptional activity. However, none of the presently available inhibitors appears to disrupt the HIF-1 pathway as their exclusive target [154]. Furthermore, their selectivity against other HIF-α subunits (HIF-2 and HIF-3 isoforms) is mostly unknown, as well as their ability to control the HIF switch. However, in recent years an effort has been made to develop selective HIF-2 inhibitors that are now in clinical studies [155]. Chronic hypoxia is a major clinical concern in the cancer field, and the potential of HIF-2-directed therapies is now possible, especially given the recent studies that indicate that tubulin beta-3 chain (*TUBB3*), which is involved in cancer progression and chemotherapy, is a HIF-2 target gene [156]. In contrast, the stimulation of the protective HIF response could be applied during the pathologies of ischemic/hypoxic and during inflammatory conditions. The many compounds proposed to induce HIF signaling target mainly VHL, PHD2 and PHD3. The most advanced, based on clinical development are the PHD3 inhibitors [157–159]. However, recent studies indicate that both FIH1 and PHDs, besides being involved in HIF response, regulate a wide range of other cellular metabolic activities [160], and this needs to be considered in order to minimize their possible side effects.

Although in the cancer field, HIF-specific inhibitors (mainly HIF-2) may open novel therapeutic approaches, their use in cardiovascular therapies is limited, given that the goal is to increase or sustain HIF activity (both HIF-1 and HIF-2) to help minimize hypoxic/ischemic tissue damage and to contribute to tissue recovery. Therefore, the design of more specific HIF switch targeting agents, including miRNA-based approaches, should be the focus of future research efforts.

## Prospects and predictions

The development of novel miRNA-based therapies for human pathologies is now of great interest. Although we can modulate the cellular miRNA levels (either with their analogs/agomiRs (miRNA overexpression) or inhibitors/antagomiRs (miRNA reduction), the number of other miRNA target genes remains a major limitation for such a strategy (Fig. 3). Another approach relies on binding of all mature miRNAs by stably overexpressing a mRNA with multiple miRNA binding sites. Consequently, the miRNAs bind this ectopic transcript rather than its endogenous target. Given that the ectopic mRNAs are used to soak up the mature miRNAs, these mRNAs have been referred to as miRNA sponges [161]. To date, several antagomiR and agomiR (mimic)-based miRNA therapeutics are currently in development [162]. However, since a single miRNA can regulate hundreds of different mRNAs, alterations in this miRNA levels will have wide-ranging and unanticipated consequences on cell metabolism. The unanticipated consequences may be caused by secondary targets that are often difficult to predict [163]. Hence, the possibilities of therapy based just on inhibition or overexpression of a specific miRNA poses a number of challenges and concerns.

**Fig. 4:**
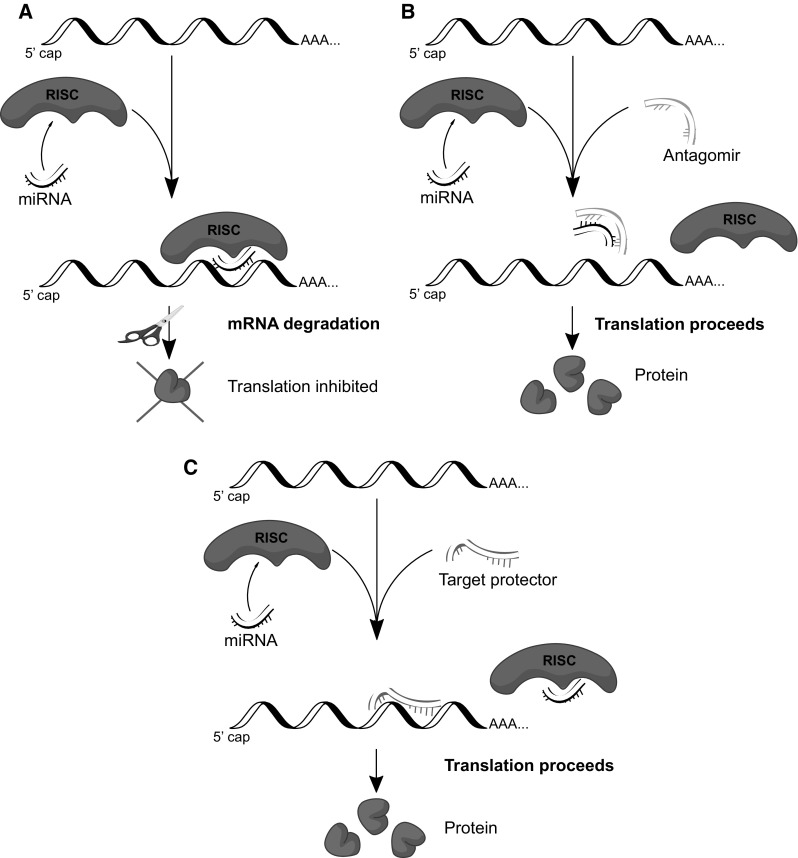
Potential miRNA therapeutic strategies. **a** agomiRs—exogenous miRNA overexpression impairs translation and leads to decreased stability of the majority of the target mRNAs. **b** antagomiRs—reduction of endogenous miRNA levels leads to increased stability and translation of the majority of the target mRNAs **c**. Target protector prevents miRNA binding to a defined specific target mRNA, leading to increased stability and translation of this mRNA (without any impact on other potential mRNA targets)

An alternate and more specific approach relies on the inhibition of the miRNA binding to a defined and unique seed sequence of a specific mRNA using a target protector. Target protectors are single-stranded, modified RNAs that inhibit the interaction of the miRNA with a specific target without blocking the effects of the particular miRNA on other targets (Fig. 4). Hence, with target protectors, we can provide specificity to the individual miRNA–mRNA interaction. Importantly, target protectors increase mRNA target expression by a modest amount and only in cells where the mRNA is already expressed [163]. Finally, if a mutation creates a new miRNA binding site that results in lower expression of an important gene, as in the case of the HIF-1α/miR-199a [122] polymorphism, treatment could occur by protecting this new mutant target site [164]. Therefore, a critical feature for this strategy is to define the specific and direct miRNA–mRNA interactions, as proposed here for HIF factors in the human endothelium.

**Fig. 5:**
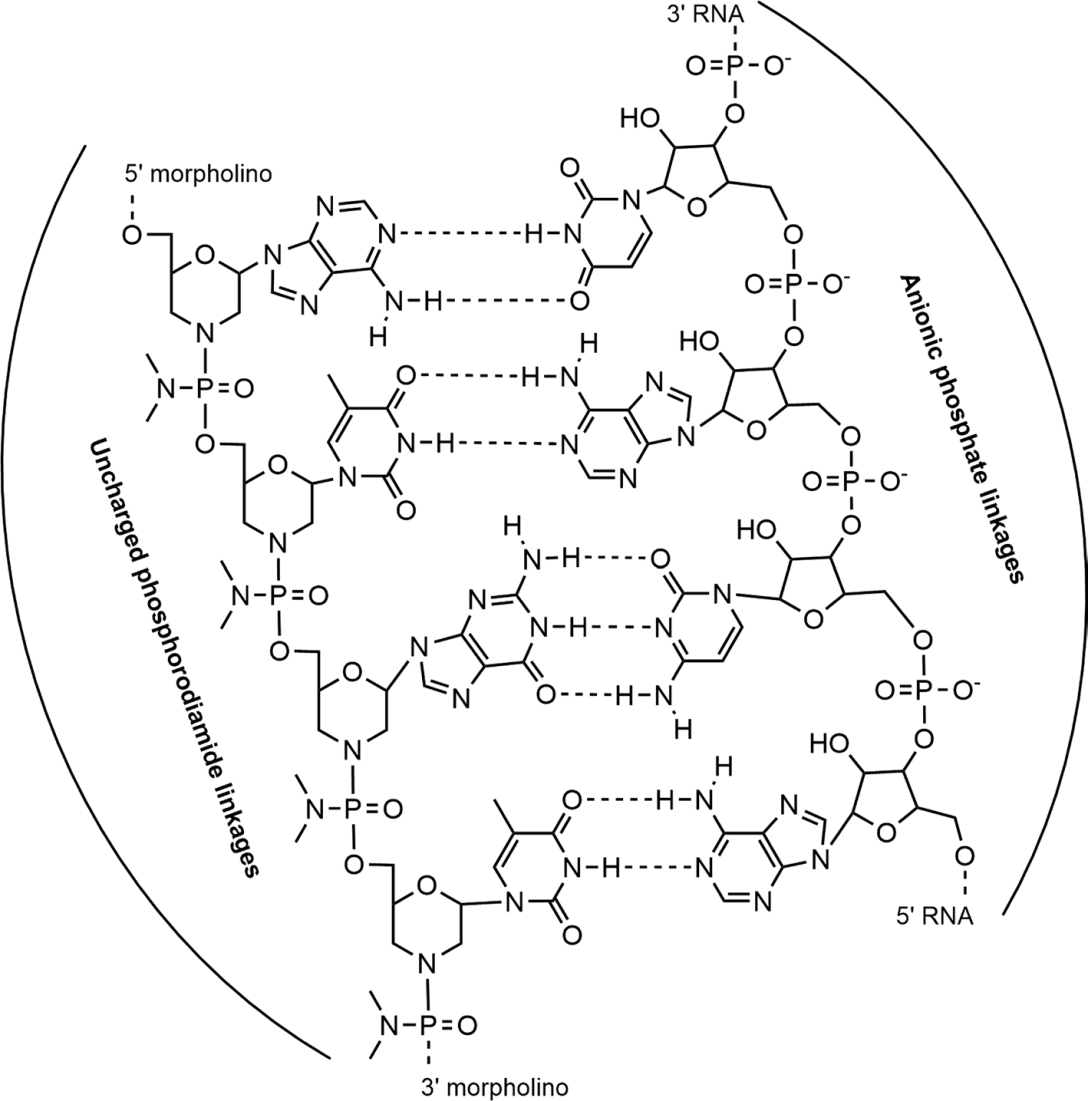
Schematic representation of the morpholino structures complementarily to the mRNA. Morpholinos have a modification of the natural RNA in which the 5-membered sugar ring is replaced by the 6-membered morpholine ring and the negatively charged phosphate intersubunit linkages are replaced by nonionic phosphorodiamidate intersubunit linkages

The target protectors technology is based on morpholinos, an oligomer molecule with a molecular structure that is a modification of natural nucleic acid in which the 5-membered sugar ring is replaced by the 6-membered morpholine ring [165] (Fig. 5). Furthermore, the negatively charged phosphate intersubunit linkages of DNA and RNA have been replaced by nonionic phosphorodiamidate intersubunit linkages [165]. With these modifications, the morpholinos are very stable in biological systems [166], show excellent solubility in aqueous solutions (typically in excess of 100 mg/ml), and have very high affinity for their complementary RNA sequences [167]. Morpholinos block access of other molecules to small (~ 25 base) specific sequences of the base-pairing surfaces of RNA (Fig. 3) [165]. In order to bind to target sequences, morpholinos require at least 14–15 contiguous bases, which makes them target-sequence-specific [165]. Furthermore, the morpholinos are free of off-target effects since they cannot interact electrostatically with proteins because of their unnatural backbone structure [165]. More importantly, the lack of a backbone charge also allows for simple and efficient delivery of morpholinos into cells by non-toxic endocytosis-assisted delivery reagents, both in vitro and in vivo [168]. The modified backbone of these oligos also prevents them from being loaded into the RISC and triggering an RNAi response [163]. This approach provides a mechanism for blocking the miRNA-mediated suppression of a specific target mRNA. Despite all these encouraging features of target protectors, the morpholinos have several potential limitations: (1) if the target sequence is not unique in the genome, potential off-target effects can occur; (2) morpholinos are generally only active for the first 5 days after delivery; and (3) they can cause nonspecific toxic effects [163].

It is clear that understanding the cellular pathways that regulate the HIF switch during hypoxia is a “Holy Grail” of vascular disorder therapies. Although the role of miRNAs in regulating the HIF-1 pathway and angiogenesis has been extensively studied, especially in cancer cells, there is limited information regarding the miRNA’s role in regulating the HIF switch in normal human endothelia. Importantly, our knowledge regarding the function of the specific HIF-α isoforms is very limited, especially regarding HIF-3 [169]. Nevertheless, the HIF switch remains a critical therapeutic target for both vascular and cancer therapies. Therapy to inhibit tumor growth is critically dependent upon the inhibition of HIF-related angiogenesis and survival (especially HIF-2), whereas therapy during stroke or myocardial infarction strongly relies on the enhancement of HIF-1 activity. The development of precise therapeutic regulation of the HIF switch is clearly important for both of these opposing actions. Hence, although many important questions regarding miRNAs biological function still remain to be answered, the possibility that miRNAs or their specific target protectors can be used in future therapeutic approaches to regulate HIF function emphasizes the importance of the studies discussed here.

## Electronic supplementary material

Below is the link to the electronic supplementary material.

Supplementary material 1 (DOCX 137 kb)

## Abbreviations

*ARNT*: Aryl hydrocarbon receptor nuclear translocator
*CDKN1A*: p21—cyclin-dependent kinase inhibitor 1
*CUL2*: Cullin-2
*DICER1*: Endoribonuclease Dicer
*EFNA3*: Ephrin A3
*EGLN1*: Prolyl hydroxylase domain-containing protein 2 (PHD2)
*ELK3*: ETS domain-containing protein Elk-3
*EPAS1*: Endothelial PAS domain-containing protein 1 (also known as hypoxia-inducible factor-2alpha HIF-2alpha)
*FBXW7*: F-box/WD repeat-containing protein 7
*GPD1L*: Glycerol-3-phosphate dehydrogenase 1-like
*HIF1A*: Hypoxia-inducible factor 1-alpha
*HIF1AN*: Hypoxia-inducible factor 1-alpha inhibitor
*HIF3A*: Hypoxia-inducible factor 3 alpha
*HMOX1*: Heme oxygenase (decycling) 1
*KLF2*: Kruppel-like factor 2
*IDH2*: Isocitrate dehydrogenase (NADP)
*IRS*-*1*: Insulin receptor substrate 1
*NRAS*: Neuroblastoma RAS viral oncogene homolog
*PDCD4*: Programmed cell death protein 4
*PDK4*: Pyruvate dehydrogenase lipoamide kinase isozyme 4
*PTEN*: Phosphatase and tensin homolog
*RPS6KB1*: p70S6 kinase
*SIRT1*: Sirtuin 1
*STAT3*: Signal transducer and activator of transcription 3
*VEGFA*: Vascular endothelial growth factor
*YWHAZ*: 14-3-3 Protein zeta/delta
