# Supplementary material for: miRNAs regulate the HIF switch during hypoxia: a novel therapeutic target
Source: Angiogenesis. 2018 Jan 27;21(2):183–202. doi: 10.1007/s10456-018-9600-2 (PMC5878208; doi:10.1007/s10456-018-9600-2)
Supplement: Supplementary file 1 — Supplementary material 1 (DOCX 137 kb) [file 10456_2018_9600_MOESM1_ESM.docx]

| **Supplemental Table 1.** Micro-RNAs involved in regulating HIFs and HIF regulatory gene levels. miRNAs proven to directly bind HIF mRNAs are in bold, and indirect effects are marked with "*". | | | | | |
| --- | --- | --- | --- | --- | --- |
| miRNA | Cell type | Impact of hypoxia on miRNA expression | miRNA target(s) (direct or indirect*) | Investigated processes | References |
| **miR-17** | pulmonary artery smooth muscle cells - PASMCs | upregulated | *EGLN1*  *HIF1A** | proliferation | [[1](#_ENREF_1)] |
|  | primary human macrophages | not shown | *HIF1A*  *EPAS1* | differentiation | [[2](#_ENREF_2)] |
|  | tumour associate macrophages | not shown | *EPAS1* | angiogenesis | [[3](#_ENREF_3)] |
| **miR-18a** | gastric carcinoma MGC-803 and HGC-27 cells | downregulated | *HIF1A* | apoptosis and invasion | [[4](#_ENREF_4)] |
|  | choroidal endothelial cells | downregulated | *HIF1A* | proliferation and migration | [[5](#_ENREF_5)] |
|  | orthotopic metastatic breast cancer xenograft model -MDA-MB-231 cells | not shown | *HIF1A* | metastasis | [[6](#_ENREF_6)] |
| **miR-20a** | primary human macrophages | not shown | *HIF1A*  *EPAS1* | differentiation | [[2](#_ENREF_2)] |
|  | tumour associate macrophages | not shown | *EPAS1* | angiogenesis | [[3](#_ENREF_3)] |
| **miR-20b** | murine tumor cell liver cancer H22, breast cancer 4T1,  prostate cancer RM1 melanoma B16 | downregulated | *HIF1A*  *VEGF* | hypoxia | [[7](#_ENREF_7)] |
|  | hepatocellular aarcinoma HepG2 cells | downregulated | *HIF1A*  *VEGF* | invasion | [[8](#_ENREF_8)] |
| miR-21 | human renal proximal tubular HK-2 cells | upregulated | *EGLN1*  *HIF1A** | ischemia/reperfusion | [[9](#_ENREF_9)] |
|  | umbilical cord blood-derived mesenchymal stem UCBMSCs cells | upregulated | *CHIP*  *HIF1A** | angiogenesis | [[10](#_ENREF_10)] |
| miR-31 | head and neck squamous cell carcinoma - HNSCC | not shown | *HIF1AN*  *HIF1A** | tumour development | [[11](#_ENREF_11)] |
| **miR-33a** | melanomas i.e. A375 cell line | not shown | *HIF1A* | proliferation invasion metastasis | [[12](#_ENREF_12)] |
| **miR-33b** | osteosarcoma U2OS cells | not shown | *HIF1A* | proliferation migration | [[13](#_ENREF_13)] |
| miR-103 | PASMCs | downregulated | *ARNT* | proliferation | [[14](#_ENREF_14)] |
| miR-107 | PASMCs | downregulated | *ARNT* | proliferation | [[14](#_ENREF_14)] |
|  | colon cancer HCT116 cells | not shown | *ARNT* | angiogenesis | [[15](#_ENREF_15)] |
|  | endothelial progenitor cells - EPCs | upregulated | *ARNT* | differentiation | [[16](#_ENREF_16)] |
| miR-135b | multiple myeloma RPMI8226 cells and HUVEC cells | upregulated | *HIF1AN*  *HIF1A** | angiogenesis | [[17](#_ENREF_17)] |
| **miR-138** | clear cell renal cell carcinoma 786-O cells | not shown | *HIF1A* | apoptosis migration | [[18](#_ENREF_18)] |
|  | human ovarian cancer SKOV-3, TOV-112D cells | not shown | *HIF1A*  *SOX4* | Invasion metastasis | [[19](#_ENREF_19)] |
| **miR-142** | pancreatic  cancer PANC-1,  SW1990, Hup, CFPAC-1 cells | downregulated | *HIF1A* | proliferation invasion | [[20](#_ENREF_20)] |
| **miR-145** | human colon cancer cells SW1116, SW480 cells | not shown | *RPS6KB1*  *HIF1A** | growth angiogenesis | [[21](#_ENREF_21)] |
|  | colorectal cancer SW1116 cells, ovarian  cancer OVCAR-3, A2780 cells | not shown | *NRAS IRS-1 HIF1A** | cancer progression | [[22](#_ENREF_22)] |
|  | neuroblastoma IMR32, SK-N-AS, SH-SY5Y, SK-N-SH cells | not shown | *EPAS1* | growth metastasis angiogenesis | [[23](#_ENREF_23)] |
| **miR-147a** | HeLa cells | upregulated | *HIF3A*  *HIF1A** | proliferation | [[24](#_ENREF_24)] |
| **miR-155** | mouse skin endothelial SENDs cells - and HUVECs | upregulated | *ELK3*  *HIF1A** | angiogenesis  migration | [[25](#_ENREF_25)] |
|  | HeLa and CaCo-2 cells | upregulated | *HIF1A* | hypoxia | [[26](#_ENREF_26)] |
| **miR-182** | human mammary epithelial  H184B5F5/M10 cells;  breast cancer MCF-7 cells | not shown | *FBXW7*  *HIF1A** | proliferation and invasion | [[27](#_ENREF_27)] |
|  | human clear cell renal cell carcinoma 786-O, OS-RC-2, Caki-1 ccRCC cells | not shown | *EPAS1*  *DICER1** | cancer progression | [[28](#_ENREF_28)] |
|  | prostate cancer PC-3, DU145 cells | upregulated | *EGLN1 HIF1AN HIF1A** | hypoxia | [[29](#_ENREF_29)] |
| miR-183 | human glioma U251, U87MG, A172, T98G, SF126 cells | not shown | *IDH2*  *HIF1A** | cancer | [[30](#_ENREF_30)] |
| **miR-186** | gastric cancer MKN45; SGC7901 cells | not shown | *HIF1A* | glycolysis | [[31](#_ENREF_31)] |
| **miR-195** | mouse chondrogenic ATDC5 cells | downregulated | *HIF1A* | apoptosis | [[32](#_ENREF_32)] |
| **miR-199a** | rat neonatal cardiomyocytes | downregulated | *HIF1A* | hypoxia and apoptosis | [[33](#_ENREF_33)] |
|  | Endometrial stromal cells; endothelial EA.hy926 cells | not shown | *HIF1A VEGFA* | angiogenesis | [[34](#_ENREF_34)] |
| miR-200b | C57BL/6 mice | upregulated | *EGLN1*  *HIF1A** | ischemic preconditionig and focal cerebral ischemia | [[35](#_ENREF_35)] |
| miR-200c | C57BL/6 mice | upregulated | *EGLN1*  *HIF1A** | ischemic preconditionig and focal cerebral ischemia | [[35](#_ENREF_35)] |
| **miR-206** | rat pulmonary artery smooth muscle cells | downregulated | *HIF1A* | hypoxia and hypertension | [[36](#_ENREF_36)] |
|  | human non-small cell lung carcinoma A59, NCI-H520 cells | downregulated | *YWHAZ*  *HIF1A** | growth and angiogenesis | [[37](#_ENREF_37)] |
| **miR-210** | HUVECs and  osteosarcoma U2OS cells | upregulated | *EFNA3* | hypoxia | [[38](#_ENREF_38)] |
|  | osteoarthritis and normal chondrocytes | not shown | *HIF3A* | proliferation | [[39](#_ENREF_39)] |
|  | human hepatocellular carcinoma SMMC-7721, PLC/PRF/5, MHCC-97L, BEL-7402 cells | upregulated | *HIF3A HIF1A** | metastasis | [[40](#_ENREF_40)] |
|  | human embryonic kidney HEK-293A cells osteosarcoma U2OS cells | upregulated | *GPD1L*  *HIF1A** | hypoxia | [[41](#_ENREF_41)] |
|  | soft tissue sarcoma cell lines | upregulated | *HIF3A* | hypoxia | [[42](#_ENREF_42)] |
|  | human synovial  fibroblasts | not shown | *GPD1L*  *HIF1A** | angiogenesis | [[43](#_ENREF_43)] |
| miR-211 | melanoma A375, WM1552C cells  and human epidermal melanocyte HEM-l cells | downregulated/ not changed | *PDK4*  *HIF1A** | metabolism | [[44](#_ENREF_44)] |
| **miR-335** | male Wistar rats and primary cultures of cortical neurons from E15 Swiss albino mouse | downregulated | *HIF1A* | hypoxia and cell death | [[45](#_ENREF_45)] |
| **miR-338-3p** | nasopharyngeal cancer cells, human immortalized nasopharyngeal epithelial cells, normal nasopharyngeal epithelial cells | not shown | HIF1A | migration and proliferation | [[46](#_ENREF_46)] |
|  | human hepatoma HepG2, SMMC-7721, BEK-7402, Hep3B,  Huh-7cells and  liver L02 cells | not shown | *HIF1A* | tumour growth | [[47](#_ENREF_47)] |
| **miR-338-5p** | male C57BL/6 mice | not shown | *HIF1A* | pulmonary  hypertension-like injures | [[48](#_ENREF_48)] |
| **miR-374b** | prostate cancer PC-3 cells | downregulated | *HIF1A*  *EPAS1* | angiogenesis | [[49](#_ENREF_49)] |
| **miR-424** | HUVECs, human MVECs, human BOECs, and MBECs | upregulated | *CUL2*  *HIF1A** | angiogenesis | [[50](#_ENREF_50)] |
|  | HUVECs | upregulated | *HIF1A* | hypoxia | [[51](#_ENREF_51)] |
|  | HUVECs | upregulated | *HIF1A*  *HIF3A* | hypoxia | [[52](#_ENREF_52)] |
| **miR-433** | primary hippocampal neurons and HUVECs | downregulated | *HIF1A* | proliferation and migration | [[53](#_ENREF_53)] |
| **miR-485-5p** | soft tissue sarcoma cells, fibrosarcoma cells, liposarcoma cells, leiomyosarcoma  cells, synovial sarcoma cells, rhabdomyosarcoma cells | upregulated | *HIF3A* | hypoxia | [[42](#_ENREF_42)] |
| miR-494 | human hepatic L02 cells | upregulated | *HIF1A** | hypoxia and apoptosis | [[54](#_ENREF_54)] |
| miR-497 | human breast cancer MCF-7 cells | downregulated | *HIF1A**  *VEGF** | angiogenesis | [[55](#_ENREF_55)] |
| **miR-519c** | lung adenocarcinoma CL1-0 and CL1-5 cells | not shown | *HIF1A* | angiogenesis | [[56](#_ENREF_56)] |
| **miR-526b-3p** | human colon cancer HT-29 and SW480 cells | not shown | *HIF1A* | cancer development and progression | [[57](#_ENREF_57)] |
| **miR-622** | human lung cancer A549 and H1299 cells | not shown | *HIF1A* | metastasis | [[58](#_ENREF_58)] |
| miR-675-5p | Human colon cancer SW480 and SW620 cells | not shown | *DDB2*  *HIF1A** | hypoxia and epithelial to mesenchymal transition | [[59](#_ENREF_59)] |
| **miR-3195** | prostate cancer PC-3 cells | not changed | *HIF1A*  *EPAS1* | angiogenesis | [[49](#_ENREF_49)] |

***ARNT*** - aryl hydrocarbon receptor nuclear translocator; ***CDKN1A*** - p21 - cyclin-dependent kinase inhibitor 1; ***CUL2*** - cullin-2; ***DICER1*** - endoribonuclease Dicer; ***EFNA3*** - ephrin A3; ***EGLN1*** - prolyl hydroxylase domain-containing protein 2 (PHD2); ***ELK3*** - ETS domain-containing protein Elk-3; ***EPAS1*** - endothelial PAS domain-containing protein 1 (also known as hypoxia-inducible factor-2alpha HIF-2alpha); ***FBXW7*** - F-box/WD repeat-containing protein 7; ***GPD1L*** - glycerol-3-phosphate dehydrogenase 1-like; ***HIF1A*** - hypoxia-inducible factor 1-alpha; ***HIF1AN*** - hypoxia-inducible factor 1-alpha inhibitor; ***HIF3A*** - hypoxia-inducible factor 3 alpha; ***HMOX1*** - heme oxygenase (decycling) 1; ***KLF2*** - kruppel like factor 2; ***IDH2*** - isocitrate dehydrogenase [NADP]; ***IRS-1*** - insulin receptor substrate 1; ***NRAS*** - neuroblastoma RAS viral oncogene homolog; ***PDCD4*** - programmed cell death protein 4; ***PDK4*** - pyruvate dehydrogenase lipoamide kinase isozyme 4; ***PTEN*** - phosphatase and tensin homolog; ***RPS6KB1*** - p70S6 kinase;***SIRT1*** - sirtuin 1; ***STAT3*** - signal transducer and activator of transcription 3; ***VEGFA*** - vascular endothelial growth factor; ***YWHAZ*** *-* 14-3-3 protein zeta/delta;

**References**

1. Chen T, Zhou Q, Tang H, Bozkanat M, Yuan JX, Raj JU, Zhou G (2016) miR-17/20 Controls Prolyl Hydroxylase 2 (PHD2)/Hypoxia-Inducible Factor 1 (HIF1) to Regulate Pulmonary Artery Smooth Muscle Cell Proliferation. Journal of the American Heart Association 5 (12). doi:10.1161/JAHA.116.004510

2. Poitz DM, Augstein A, Gradehand C, Ende G, Schmeisser A, Strasser RH (2013) Regulation of the Hif-system by micro-RNA 17 and 20a - role during monocyte-to-macrophage differentiation. Molecular immunology 56 (4):442-451. doi:10.1016/j.molimm.2013.06.014

3. Xu Z, Zhao L, Zhu LY, He M, Zheng L, Wu Y (2013) MicroRNA-17, 20a regulates the proangiogenic function of tumor-associated macrophages via targeting hypoxia-inducible factor 2alpha. PloS one 8 (10):e77890. doi:10.1371/journal.pone.0077890

4. Wu F, Huang W, Wang X (2015) microRNA-18a regulates gastric carcinoma cell apoptosis and invasion by suppressing hypoxia-inducible factor-1alpha expression. Experimental and therapeutic medicine 10 (2):717-722. doi:10.3892/etm.2015.2546

5. Han F, Wu Y, Jiang W (2015) MicroRNA-18a Decreases Choroidal Endothelial Cell Proliferation and Migration by Inhibiting HIF1A Expression. Medical science monitor : international medical journal of experimental and clinical research 21:1642-1647. doi:10.12659/MSM.893068

6. Krutilina R, Sun W, Sethuraman A, Brown M, Seagroves TN, Pfeffer LM, Ignatova T, Fan M (2014) MicroRNA-18a inhibits hypoxia-inducible factor 1alpha activity and lung metastasis in basal breast cancers. Breast cancer research : BCR 16 (4):R78. doi:10.1186/bcr3693

7. Lei Z, Li B, Yang Z, Fang H, Zhang GM, Feng ZH, Huang B (2009) Regulation of HIF-1alpha and VEGF by miR-20b tunes tumor cells to adapt to the alteration of oxygen concentration. PloS one 4 (10):e7629. doi:10.1371/journal.pone.0007629

8. Xue TM, Tao LD, Zhang M, Zhang J, Liu X, Chen GF, Zhu YJ, Zhang PJ (2015) Clinicopathological Significance of MicroRNA-20b Expression in Hepatocellular Carcinoma and Regulation of HIF-1alpha and VEGF Effect on Cell Biological Behaviour. Disease markers 2015:325176. doi:10.1155/2015/325176

9. Jiao X, Xu X, Teng J, Fang Y, Zhang H, Liang M, Ding X (2016) miR-21 contributes to renal protection by targeting prolyl hydroxylase domain protein 2 in delayed ischemic preconditioning. Nephrology (Carlton). doi:10.1111/nep.12787

10. Zhou Y, Zhu Y, Zhang L, Wu T, Zhang W, Decker AM, He J, Liu J, Wu Y, Jiang X, Zhang Z, Liang C, Zou D (2016) Human Stem Cells Overexpressing miR-21 Promote Angiogenesis in Critical Limb Ischemia by Targeting CHIP to Enhance HIF-1alpha Activity. Stem Cells 34 (4):924-934. doi:10.1002/stem.2321

11. Liu CJ, Tsai MM, Hung PS, Kao SY, Liu TY, Wu KJ, Chiou SH, Lin SC, Chang KW (2010) miR-31 ablates expression of the HIF regulatory factor FIH to activate the HIF pathway in head and neck carcinoma. Cancer research 70 (4):1635-1644. doi:10.1158/0008-5472.CAN-09-2291

12. Zhou J, Xu D, Xie H, Tang J, Liu R, Li J, Wang S, Chen X, Su J, Zhou X, Xia K, He Q, Chen J, Xiong W, Cao P, Cao K (2015) miR-33a functions as a tumor suppressor in melanoma by targeting HIF-1alpha. Cancer Biol Ther 16 (6):846-855. doi:10.1080/15384047.2015.1030545

13. Zhou Y, Yang C, Wang K, Liu X, Liu Q (2016) MicroRNA-33b inhibits the proliferation and migration of osteosarcoma cells via targeting hypoxia-inducible factor-1alpha. Oncology research. doi:10.3727/096504016X14743337535446

14. Deng B, Du J, Hu R, Wang AP, Wu WH, Hu CP, Li YJ, Li XH (2016) MicroRNA-103/107 is involved in hypoxia-induced proliferation of pulmonary arterial smooth muscle cells by targeting HIF-1 beta. Life Sci 147:117-124. doi:10.1016/j.lfs.2016.01.043

15. Yamakuchi M, Lotterman CD, Bao C, Hruban RH, Karim B, Mendell JT, Huso D, Lowenstein CJ (2010) P53-induced microRNA-107 inhibits HIF-1 and tumor angiogenesis. Proceedings of the National Academy of Sciences of the United States of America 107 (14):6334-6339. doi:10.1073/pnas.0911082107

16. Meng S, Cao J, Wang L, Zhou Q, Li Y, Shen C, Zhang X, Wang C (2012) MicroRNA 107 partly inhibits endothelial progenitor cells differentiation via HIF-1beta. PloS one 7 (7):e40323. doi:10.1371/journal.pone.0040323

17. Umezu T, Tadokoro H, Azuma K, Yoshizawa S, Ohyashiki K, Ohyashiki JH (2014) Exosomal miR-135b shed from hypoxic multiple myeloma cells enhances angiogenesis by targeting factor-inhibiting HIF-1. Blood 124 (25):3748-3757. doi:10.1182/blood-2014-05-576116

18. Song T, Zhang X, Wang C, Wu Y, Cai W, Gao J, Hong B (2011) MiR-138 suppresses expression of hypoxia-inducible factor 1alpha (HIF-1alpha) in clear cell renal cell carcinoma 786-O cells. Asian Pacific journal of cancer prevention : APJCP 12 (5):1307-1311

19. Yeh YM, Chuang CM, Chao KC, Wang LH (2013) MicroRNA-138 suppresses ovarian cancer cell invasion and metastasis by targeting SOX4 and HIF-1alpha. International journal of cancer 133 (4):867-878. doi:10.1002/ijc.28086

20. Lu Y, Ji N, Wei W, Sun W, Gong X, Wang X (2017) MiR-142 modulates human pancreatic cancer proliferation and invasion by targeting hypoxia-inducible factor 1 (HIF-1alpha) in the tumor microenvironments. Biology open 6 (2):252-259. doi:10.1242/bio.021774

21. Xu Q, Liu LZ, Qian X, Chen Q, Jiang Y, Li D, Lai L, Jiang BH (2012) MiR-145 directly targets p70S6K1 in cancer cells to inhibit tumor growth and angiogenesis. Nucleic acids research 40 (2):761-774. doi:10.1093/nar/gkr730

22. Yin Y, Yan ZP, Lu NN, Xu Q, He J, Qian X, Yu J, Guan X, Jiang BH, Liu LZ (2013) Downregulation of miR-145 associated with cancer progression and VEGF transcriptional activation by targeting N-RAS and IRS1. Biochimica et biophysica acta 1829 (2):239-247. doi:10.1016/j.bbagrm.2012.11.006

23. Zhang H, Pu J, Qi T, Qi M, Yang C, Li S, Huang K, Zheng L, Tong Q (2014) MicroRNA-145 inhibits the growth, invasion, metastasis and angiogenesis of neuroblastoma cells through targeting hypoxia-inducible factor 2 alpha. Oncogene 33 (3):387-397. doi:10.1038/onc.2012.574

24. Wang F, Zhang H, Xu N, Huang N, Tian C, Ye A, Hu G, He J, Zhang Y (2016) A novel hypoxia-induced miR-147a regulates cell proliferation through a positive feedback loop of stabilizing HIF-1alpha. Cancer Biol Ther 17 (8):790-798. doi:10.1080/15384047.2016.1195040

25. Robertson ED, Wasylyk C, Ye T, Jung AC, Wasylyk B (2014) The oncogenic MicroRNA Hsa-miR-155-5p targets the transcription factor ELK3 and links it to the hypoxia response. PloS one 9 (11):e113050. doi:10.1371/journal.pone.0113050

26. Bruning U, Cerone L, Neufeld Z, Fitzpatrick SF, Cheong A, Scholz CC, Simpson DA, Leonard MO, Tambuwala MM, Cummins EP, Taylor CT (2011) MicroRNA-155 promotes resolution of hypoxia-inducible factor 1alpha activity during prolonged hypoxia. Molecular and cellular biology 31 (19):4087-4096. doi:10.1128/MCB.01276-10

27. Chiang CH, Chu PY, Hou MF, Hung WC (2016) MiR-182 promotes proliferation and invasion and elevates the HIF-1alpha-VEGF-A axis in breast cancer cells by targeting FBXW7. American journal of cancer research 6 (8):1785-1798

28. Fan Y, Li H, Ma X, Gao Y, Bao X, Du Q, Ma M, Liu K, Yao Y, Huang Q, Zhang Y, Zhang X (2016) Dicer suppresses the malignant phenotype in VHL-deficient clear cell renal cell carcinoma by inhibiting HIF-2alpha. Oncotarget 7 (14):18280-18294. doi:10.18632/oncotarget.7807

29. Li Y, Zhang D, Wang X, Yao X, Ye C, Zhang S, Wang H, Chang C, Xia H, Wang YC, Fang J, Yan J, Ying H (2015) Hypoxia-inducible miR-182 enhances HIF1alpha signaling via targeting PHD2 and FIH1 in prostate cancer. Scientific reports 5:12495. doi:10.1038/srep12495

30. Tanaka H, Sasayama T, Tanaka K, Nakamizo S, Nishihara M, Mizukawa K, Kohta M, Koyama J, Miyake S, Taniguchi M, Hosoda K, Kohmura E (2013) MicroRNA-183 upregulates HIF-1alpha by targeting isocitrate dehydrogenase 2 (IDH2) in glioma cells. Journal of neuro-oncology 111 (3):273-283. doi:10.1007/s11060-012-1027-9

31. Liu L, Wang Y, Bai R, Yang K, Tian Z (2016) MiR-186 inhibited aerobic glycolysis in gastric cancer via HIF-1alpha regulation. Oncogenesis 5:e224. doi:10.1038/oncsis.2016.35

32. Bai R, Zhao AQ, Zhao ZQ, Liu WL, Jian DM (2015) MicroRNA-195 induced apoptosis in hypoxic chondrocytes by targeting hypoxia-inducible factor 1 alpha. European review for medical and pharmacological sciences 19 (4):545-551

33. Rane S, He M, Sayed D, Vashistha H, Malhotra A, Sadoshima J, Vatner DE, Vatner SF, Abdellatif M (2009) Downregulation of miR-199a derepresses hypoxia-inducible factor-1alpha and Sirtuin 1 and recapitulates hypoxia preconditioning in cardiac myocytes. Circulation research 104 (7):879-886. doi:10.1161/CIRCRESAHA.108.193102

34. Dai L, Lou W, Zhu J, Zhou X, Di W (2015) MiR-199a inhibits the angiogenic potential of endometrial stromal cells under hypoxia by targeting HIF-1alpha/VEGF pathway. International journal of clinical and experimental pathology 8 (5):4735-4744

35. Lee ST, Chu K, Jung KH, Yoon HJ, Jeon D, Kang KM, Park KH, Bae EK, Kim M, Lee SK, Roh JK (2010) MicroRNAs induced during ischemic preconditioning. Stroke 41 (8):1646-1651. doi:10.1161/STROKEAHA.110.579649

36. Yue J, Guan J, Wang X, Zhang L, Yang Z, Ao Q, Deng Y, Zhu P, Wang G (2013) MicroRNA-206 is involved in hypoxia-induced pulmonary hypertension through targeting of the HIF-1alpha/Fhl-1 pathway. Laboratory investigation; a journal of technical methods and pathology 93 (7):748-759. doi:10.1038/labinvest.2013.63

37. Xue D, Yang Y, Liu Y, Wang P, Dai Y, Liu Q, Chen L, Shen J, Ju H, Li Y, Tan Z (2016) MicroRNA-206 attenuates the growth and angiogenesis in non-small cell lung cancer cells by blocking the 14-3-3zeta/STAT3/HIF-1alpha/VEGF signaling. Oncotarget 7 (48):79805-79813. doi:10.18632/oncotarget.12972

38. Fasanaro P, D'Alessandra Y, Di Stefano V, Melchionna R, Romani S, Pompilio G, Capogrossi MC, Martelli F (2008) MicroRNA-210 modulates endothelial cell response to hypoxia and inhibits the receptor tyrosine kinase ligand Ephrin-A3. The Journal of biological chemistry 283 (23):15878-15883. doi:10.1074/jbc.M800731200

39. Li Z, Meng D, Li G, Xu J, Tian K, Li Y (2016) Overexpression of microRNA-210 promotes chondrocyte proliferation and extracellular matrix deposition by targeting HIF-3alpha in osteoarthritis. Molecular medicine reports 13 (3):2769-2776. doi:10.3892/mmr.2016.4878

40. Kai AK, Chan LK, Lo RC, Lee JM, Wong CC, Wong JC, Ng IO (2016) Down-regulation of TIMP2 by HIF-1alpha/miR-210/HIF-3alpha regulatory feedback circuit enhances cancer metastasis in hepatocellular carcinoma. Hepatology 64 (2):473-487. doi:10.1002/hep.28577

41. Kelly TJ, Souza AL, Clish CB, Puigserver P (2011) A hypoxia-induced positive feedback loop promotes hypoxia-inducible factor 1alpha stability through miR-210 suppression of glycerol-3-phosphate dehydrogenase 1-like. Molecular and cellular biology 31 (13):2696-2706. doi:10.1128/MCB.01242-10

42. Gits CM, van Kuijk PF, de Rijck JC, Muskens N, Jonkers MB, van IWF, Mathijssen RH, Verweij J, Sleijfer S, Wiemer EA (2014) MicroRNA response to hypoxic stress in soft tissue sarcoma cells: microRNA mediated regulation of HIF3alpha. BMC cancer 14:429. doi:10.1186/1471-2407-14-429

43. Liu SC, Chuang SM, Hsu CJ, Tsai CH, Wang SW, Tang CH (2014) CTGF increases vascular endothelial growth factor-dependent angiogenesis in human synovial fibroblasts by increasing miR-210 expression. Cell death & disease 5:e1485. doi:10.1038/cddis.2014.453

44. Mazar J, Qi F, Lee B, Marchica J, Govindarajan S, Shelley J, Li JL, Ray A, Perera RJ (2016) MicroRNA 211 Functions as a Metabolic Switch in Human Melanoma Cells. Molecular and cellular biology 36 (7):1090-1108. doi:10.1128/MCB.00762-15

45. Liu FJ, Kaur P, Karolina DS, Sepramaniam S, Armugam A, Wong PT, Jeyaseelan K (2015) MiR-335 Regulates Hif-1alpha to Reduce Cell Death in Both Mouse Cell Line and Rat Ischemic Models. PloS one 10 (6):e0128432. doi:10.1371/journal.pone.0128432

46. Shan Y, Li X, You B, Shi S, Zhang Q, You Y (2015) MicroRNA-338 inhibits migration and proliferation by targeting hypoxia-induced factor 1alpha in nasopharyngeal carcinoma. Oncology reports 34 (4):1943-1952. doi:10.3892/or.2015.4195

47. Xu H, Zhao L, Fang Q, Sun J, Zhang S, Zhan C, Liu S, Zhang Y (2014) MiR-338-3p inhibits hepatocarcinoma cells and sensitizes these cells to sorafenib by targeting hypoxia-induced factor 1alpha. PloS one 9 (12):e115565. doi:10.1371/journal.pone.0115565

48. Ji X, Zhang Y, Ku T, Yun Y, Li G, Sang N (2016) MicroRNA-338-5p modulates pulmonary hypertension-like injuries caused by SO2, NO2 and PM2.5 co-exposure through targeting the HIF-1[small alpha]/Fhl-1 pathway. Toxicology Research 5 (6):1548-1560. doi:10.1039/c6tx00257a

49. Sohn EJ, Won G, Lee J, Lee S, Kim SH (2015) Upregulation of miRNA3195 and miRNA374b Mediates the Anti-Angiogenic Properties of Melatonin in Hypoxic PC-3 Prostate Cancer Cells. Journal of Cancer 6 (1):19-28. doi:10.7150/jca.9591

50. Ghosh G, Subramanian IV, Adhikari N, Zhang X, Joshi HP, Basi D, Chandrashekhar YS, Hall JL, Roy S, Zeng Y, Ramakrishnan S (2010) Hypoxia-induced microRNA-424 expression in human endothelial cells regulates HIF-alpha isoforms and promotes angiogenesis. The Journal of clinical investigation 120 (11):4141-4154. doi:10.1172/JCI42980

51. Bartoszewska S, Kochan K, Piotrowski A, Kamysz W, Ochocka RJ, Collawn JF, Bartoszewski R (2015) The hypoxia-inducible miR-429 regulates hypoxia-inducible factor-1 alpha expression in human endothelial cells through a negative feedback loop. Faseb Journal 29 (4):1467-1479. doi:10.1096/fj.14-267054

52. Janaszak-Jasiecka A, Bartoszewska S, Kochan K, Piotrowski A, Kalinowski L, Kamysz W, Ochocka RJ, Bartoszewski R, Collawn JF (2016) miR-429 regulates the transition between Hypoxia-Inducible Factor (HIF)1A and HIF3A expression in human endothelial cells. Scientific reports 6:22775. doi:10.1038/srep22775

53. Zhang L, Zhang Y, Zhang X, Jiang Y, Xiao X, Tan J, Yuan W, Liu Y (2016) MicroRNA-433 Inhibits the Proliferation and Migration of HUVECs and Neurons by Targeting Hypoxia-Inducible Factor 1 Alpha. Journal of molecular neuroscience : MN. doi:10.1007/s12031-016-0853-1

54. Sun G, Zhou Y, Li H, Guo Y, Shan J, Xia M, Li Y, Li S, Long D, Feng L (2013) Over-expression of microRNA-494 up-regulates hypoxia-inducible factor-1 alpha expression via PI3K/Akt pathway and protects against hypoxia-induced apoptosis. Journal of biomedical science 20:100. doi:10.1186/1423-0127-20-100

55. Wu Z, Cai X, Huang C, Xu J, Liu A (2016) miR-497 suppresses angiogenesis in breast carcinoma by targeting HIF-1alpha. Oncology reports 35 (3):1696-1702. doi:10.3892/or.2015.4529

56. Cha ST, Chen PS, Johansson G, Chu CY, Wang MY, Jeng YM, Yu SL, Chen JS, Chang KJ, Jee SH, Tan CT, Lin MT, Kuo ML (2010) MicroRNA-519c suppresses hypoxia-inducible factor-1alpha expression and tumor angiogenesis. Cancer research 70 (7):2675-2685. doi:10.1158/0008-5472.CAN-09-2448

57. Zhang R, Zhao J, Xu J, Wang J, Jia J (2016) miR-526b-3p functions as a tumor suppressor in colon cancer by regulating HIF-1alpha. American journal of translational research 8 (6):2783-2789

58. Cheng CW, Chen PM, Hsieh YH, Weng CC, Chang CW, Yao CC, Hu LY, Wu PE, Shen CY (2015) Foxo3a-mediated overexpression of microRNA-622 suppresses tumor metastasis by repressing hypoxia-inducible factor-1alpha in ERK-responsive lung cancer. Oncotarget 6 (42):44222-44238. doi:10.18632/oncotarget.5826

59. Costa V, Lo Dico A, Rizzo A, Rajata F, Tripodi M, Alessandro R, Conigliaro A (2017) MiR-675-5p supports hypoxia induced epithelial to mesenchymal transition in colon cancer cells. Oncotarget. doi:10.18632/oncotarget.14464
